# Inhibitory Effect of PRMT5/MTA Inhibitor on MTAP‐Deficient Glioma May Be Influenced by Surrounding Normal Cells

**DOI:** 10.1002/cam4.70526

**Published:** 2024-12-23

**Authors:** Yunjie Wang, Xiaohui Sun, Runchen Ma, Xiaofan Zhang, Shengmin Ji, Zhaofeng Liu, Gangqiang Yang, Hongbo Wang, Peng Zhang, Jianzhao Zhang, Jingwei Tian

**Affiliations:** ^1^ School of Pharmacy, Key Laboratory of Molecular Pharmacology and Drug Evaluation (Yantai University), Ministry of Education, Collaborative Innovation Center of Advanced Drug Delivery System and Biotech Drugs in Universities of Shandong Yantai University Yantai Shandong People's Republic of China

**Keywords:** glioblastoma, inhibitor of PRMT5/MTA, MTAP‐deleted, synthetic lethal hypothesis

## Abstract

**Background:**

Methylthioadenosine phosphorylase (MTAP) and protein arginine methyltransferase 5 (PRMT5) are considered to be a synthetic lethal pair of targets, due to the fact that deletion of MTAP leads to massive production of methylthioadenosine (MTA) decreasing the activity of PRMT5. In vitro and in vivo experiments have demonstrated that MRTX1719, a small molecule that selectively binds PRMT5/MTA complex, significantly inhibits the proliferation of MTAP‐deficient tumors and has a weak toxic effect on normal cells. However, it has been reported that MTAP‐deleted tumors did not significantly accumulate MTA in vivo due to metabolism of MTA by MTAP‐expressing stroma, which might lead to a diminished anti‐cancer effect of MRTX1719.

**Methods:**

We first analyzed whether there were MTAP‐expressing normal intracerebral cells around MTAP‐deficient glioma tissues by paraffin‐embedded tissue microarray of human glioma specimens. Then, in vivo and in vitro models of MTAP‐deficient gliomas coexisting with neurons or glial cells were constructed for evaluating the effectiveness of the anti‐tumor effects of MRTX1719 in this setting.

**Results:**

MTAP‐deficient gliomas were surrounded by a large number of MTAP‐expressing normal cells, and the presence of these cells significantly reduced the inhibitory effect of MRTX1719 on MTAP‐deficient glioma cells in vitro and in vivo.

**Conclusions:**

Due to the complexity of the tumor environment in vivo, the anti‐tumor effects of PRMT5/MTA‐specific inhibitors may be somewhat attenuated, and their ability to achieve suitable therapeutic effects in the clinic might require more in‐depth studies.

## 1 Introduction

Glioblastoma multiforme (GBM) is the most common and deadliest brain tumor, with a median survival time of about 15 months after diagnosis and a low prognosis for recovery, with the recurrence rate approaching 90%, and with < 10% surviving beyond 5 years [1, 2]. Despite the development of cancer therapeutic strategies, surgery, radiation, and chemotherapy still account for the greatest proportion of glioma treatments which lead to no more wonderful breakthrough in the treatment of GBM [3]. Therefore, developing therapies that directly target the molecular drivers of diseases will have a great clinical impact.

Mutations in one gene sensitize cells to the suppression of another gene, which is known as synthetic lethal interactions, and have been used to develop targeted cancer therapeutics [4]. Homologous chromosomal deletion of the methylthioadenosine phosphorylase (MTAP) gene is present in approximately 50% of GBM cases, making MTAP one of the most commonly deleted genes in GBM [5]. Despite this prevalence, there are still few approaches to selectively target cyclin‐dependent kinase inhibitor 2A (CDKN2A)/MTAP‐deleted tumors [6]. Although there are cyclin‐dependent kinase 4 (CDK4)/CDK6 inhibitors used in the study of CDKN2A‐deleted cells in vitro, to date, this therapeutic method has not produced curative effect in clinical research [7]. This discovery enriches researchers' thoughts that MTAP may be used to synthesize lethal strategies. What's more, MTAP loss results in the accumulation of methylthioadenosine (MTA), which inhibits protein arginine methyltransferase 5 (PRMT5) activity and leads to reduced basal PRMT5 methylation in MTAP‐deleted cancers [8, 9]. PRMT5 regulates a number of proliferative and biosynthetic processes and has been demonstrated to be important for proliferation in tumor models in vivo [10, 11]. Inhibitors that could trap PRMT5 may have particularly robust therapeutic window in MTAP‐deleted tumors. Thus, considering that MTAP‐deleted tumors are relatively more sensitive to PRMT5 inhibition [12], synthetic lethal strategies to exploit MTAP loss with methionine starvation or by inhibiting denovo purine synthesis have been proposed.

The biological role of PRMT5‐supporting oncogenic pathways has led to the rational development of highly selective PRMT5 inhibitors [13]. Researchers believe that the vulnerability of PRMT5 in MTAP‐deficient cancers may extend both upstream of PRMT5 (methionine adenosyltransferase 2A, MAT2A) and downstream of PRMT5 (RIOK1 and other PRMT5 co‐complex members). Therapies targeting of MAT2A, RIOK1, or other PRMT5 co‐complex members selectively affect MTAP‐deleted cancers while sparing MTAP‐expressing normal tissues, which address the unmet clinical need of human cancers with the deletion of the MTAP locus [8]. Considering the high affinity of PRMT5 for MTA and a high‐MTA environment of MTAP‐deficient cells, we predict that a small molecule that selectively binds and stabilizes the PRMT5/MTA complex is predicted to further inhibit the residual PRMT5 activity in MTAP‐deleted tumors and may represent a synthetic lethal‐based precision medicine for patients [14]. However, clinical efficacy of such approaches has also not been demonstrated [15].

All this seems very reasonable, but Yasaman Barekatain and his team put forward different views. They concluded that whether this concept can be called a therapeutic target depends on whether or not significant MTA accumulation is exhibited in human glioblastoma cells [16]. However, the mammalian metabolome is characterized by a high degree of flexibility and redundancy [17]. MTA accumulates to an intracellular concentration of approximately 100 μM, and cells begin to excrete excess MTA [8]. They found that MTA secreted by MTAP‐deficient cells in vitro leads to high extracellular levels of MTA. However, in in vivo experiments, no significant MTA accumulation was found in MTAP‐deficient primary glioblastoma tumors due to the metabolism of MTA by a stroma with normal MTAP expression [16]. They concluded that there are prominent metabolic differences between in vitro tumor cells and primary human tumor tissue that must be taken into account when developing precise therapeutic strategies for homozygous MTAP‐deficient glioblastomas [16].

MRTX1719, a small molecule, binds to PRMT5 in an MTA‐cooperative manner and is designed based on PRMT5 as a synthetic lethal target for MTAP gene loss [18]. Further, it is demonstrated that cellular selectivity of MRTX1719 has > 70‐fold potency for killing MTAP‐delete cells compared with MTAP‐expressing cells [14, 19]. This new class of MTA‐cooperative PRMT5 inhibitors overcomes prior issues of drug toxicity and kills the tumor cells selectively, realizing a good application prospect. Given that MTAP deletion and consequent MTA accumulation are responsible for sensitization to the PRMT5/MTA constitutive inhibitor (MRTX1719), depletion of the accumulated MTA may result in MTAP‐deficient cells insensitive to MRTX1719. GBMs are surrounded by MTAP‐expressing cells, including neurons, microglia, astrocytes, etc., which may result in the accumulation of MTA by MTAP‐deficient GBMs being metabolized by surrounding MTAP‐expressing normal cells, and leading to the lethality of MRTX1719 against MTAP‐deficient GBMs. In such an in vivo environment, it is worth exploring whether MRTX1719 still has a killing effect on MTAP gene‐deficient GBMs. Therefore, in this study, we first analyzed the expression amount and site of MTAP and PRMT5 in the tissues of human GBMs to investigate the role of PRMT5/MTA inhibitors in the treatment of GBMs. Then, we simulated the inhibitory effect of PRMT5/MTA inhibitor on GBMs by in vitro and in vivo experiments in which MTAP‐deficient glioma cells coexisted with microglia and neurons with normal MTAP expression. Finally, we hope that this study will provide some experimental basis for the role of PRMT5/MTA inhibitors in the treatment of gliomas.

## 2 Materials and Methods

### 2.1 Reagent

Dulbecco's modified Eagle's medium (DMEM), modified Eagle's medium (MEM), and fetal bovine serum (FBS) were obtained from Gibco (Grand Island, NY, USA), penicillin–streptomycin was acquired from Beyotime (Shanghai, China), and nonessential amino acids (NEAA) were purchased from Thermo Fisher Scientific (Shanghai, China).

The following antibodies were used in the study: MTAP (Cell Signaling Technology, Danvers, MA, United States, 1:1000 dilution), PRMT5 (Proteintech, Wuhan, China, 1:1000 dilution), Iba‐1 (Cell Signaling Technology, Boston, US, 1:1000 dilution), and β‐actin (Beyotime, Shanghai, China, 1:1000 dilution).

MRTX1719 was purchased from MCE (Shanghai, China) (Purity: 99.40%).

### 2.2 Paraffin‐Embedded Tissue Microarray of Human Glioma Specimens and Patient Cohorts

Paraffin‐embedded tissue microarray from 180 patients with glioma (catalog: HBraG180Su01) was purchased from Shanghai Outdo Biotech (Shanghai, China). A total of 180 patients (112 males and 68 females, aged from 2 to 80 years) who suffered from glioma were selected. The detailed clinical parameters of these patients are shown in Supporting Information 1.

### 2.3 Multiplexed Immunofluorescence Staining and Image Analysis

Multiplexed immunofluorescence staining was carried out by using the PANO Multiplex IHC kit (Panovue) in combination with automated quantitative analysis (PerkinElmer, USA) based on the manufacturer's instructions to characterize the expressions and localization of MTAP, PRMT5, and Iba‐1 in the paraffin‐embedded tissue microarray of human glioma specimens. Staining was performed following a standard protocol involving sequential incubation with primary and secondary antibodies, followed by tyramide signal amplification (TSA). Afterward, the TissueFAXS system (TissueGnostics Asia Pacific Limited, Austria) was used to conduct the panoramic multispectral scanning of PC TMA slide, and then the acquired images were processed using StrataQuest analysis software (Version No. 7.0.1.165, TissueGnostics Asia Pacific Limited, Austria). Finally, we count the positive cells of all samples of the TMA. Information on the expression of MTAP, PRMT5, and Iba‐1 in GBM tissues are provided in Supporting Information 2.

### 2.4 Cell Culture

Three kinds of glioma cell lines (Hs683, U‐118MG, and GL261), human microglia cell line (HMC3), and human neuroblastoma cell line (SH‐SY5Y) were purchased from American Type Culture Collection (ATCC, China). All cells were incubated at 37°C (5% CO_2_). Hs683, U‐118MG, and GL261 cells were maintained in DMEM with 10% FBS and 1% penicillin–streptomycin. HMC3 cells were cultured in MEM supplemented with 10% FBS and 1% penicillin–streptomycin. SH‐SY5Y cells were cultured in MEM with10% FBS, 1% NEAA, and 1% penicillin–streptomycin.

### 2.5 Evaluation of Cell Viability

1. Cell viability was detected using a CellTiter‐Glo luminescent cell viability assay (Promega, USA) according to the manufacturer's instructions. Cells were paved in white‐bottomed 96‐well plates with 800 cells/200 μL cell suspension solution. After 12 h, the compound dissolved in DMSO was added. Cells were cultured in an incubator for 8 days, and the medium added with the compound was changed once on the fourth day. Then, 100 μL of Celltiter‐Glo solution was added and mixed well for 5 min, and the luminous intensity of each well was detected with a spectrophotometer (BioTek, Winooski, VT, United States).
2. In order to simulate the real tumor environment, cells were cultured in the modified Boyden chamber with a pore size of 0.4 μm in 24‐well plates. In the simulation of the early stage, 800 cells/200 μL MTAP^−/−^ (Hs683 or U‐118MG) cell suspension solutions were added in the upper chamber, and approximately 1000 MTAP^WT^ (HMC3 or SH‐SY5Y) cells suspended in 500 μL of the medium were seeded in the 24‐well plate. Subsequent operations are as described above. When simulating the late stage, 800 MTAP^WT^ cells were added to the upper chamber in 200 μL media, while 1000 MTAP^−/−^ cells were added to the lower chamber to a 500 μL medium. After 8 days of co‐culture, the cell viability test was carried out, and the liquid was changed once on the fourth day.

### 2.6 Colony Formation

Four kinds of cells were separately seeded in six‐well plates at 1000 cells per well and cultured for 14 days. MRTX1719 was added in a dose‐dependent manner after 24 h. Subsequently, the cells were fixed with 4% paraformaldehyde for 15 min. Finally, the cells were stained using 0.5% crystal violet for 30 min. The colonies were photographed and counted using Image J.

### 2.7 Animals

A total of 32 adult male BALB/c nude mice (initial weight 18–22 g) were purchased from Beijing Huafukang Bio‐Technology Co. Ltd and used in experiment. Four unsuccessful models were excluded, and a total of 28 models were used for formal experiments. The mice were housed in groups with a 12/12‐h light/dark cycle and were allowed to drink and eat freely. After the experiment, the body weight was measured every 3 days. All of the procedures were approved by the Institutional Animal Care and Use Committee of Yantai University, China.

### 2.8 Tumor Xenograft Experiments

Hs683 cells (1 × 10^7^) and a mixture of cells (Hs683 and HMC3 are 5 × 10^6^ respectively) were implanted into the back of mice by subcutaneous injection. The tumor volume was measured every other day until it reached 100 cubic millimeters. Mice were randomly divided into the following groups: Vehicle‐1 (Hs683), Vehicle‐2 (Hs683 and HMC3), MRTX1719‐1 (Hs683), and MRTX1719‐2 (Hs683 and HMC3). MRTX1719 was given dosing by intraperitoneal injection (0.2 mL/20 g) at a dose of 10 mg/kg (1% DMSO, 10% Solutol and 89% saline) every day. The vehicle group was given an equal volume solvent. Tumor growth was measured every 3 days during treatment. Finally, the mice were sacrificed with CO_2_, and tumors were removed for comparison.

### 2.9 Western Blot

Total protein from cells and tumor tissues were collected and homogenized in RIPA buffer. The sample was electrophoresed on 10% SDS–polyacrylamide gels and then transferred to a PVDF membrane The primary antibodies MTAP, PRMT5, and β‐actin were used for Western blot and incubated overnight at 4°C, and secondary antibodies were incubated at room temperature for 1 h. Imaging was performed using Image Quant LAS4000 (GE, United States).

### 2.10 LC–MS/MS Analysis

Male C57BL/6 mice were injected intraperitoneally with MRTX1719; blood and brain tissues were collected 15 min, 1 h, 2 h, 4 h, and 10 h after drug administration. Samples were measured by a Shimadzu chromatography system (Shimadzu, Kyoto, Japan) coupled with an AB Sciex ESI triple–quadrupole 4500 mass spectrometer (SCIEX Triple Quad 4500 LC–MS/MS, Applied Biosystems Sciex, Ontario, Canada) using Analyst 1.4.2 software. Further, an HPLC column (ACQUITY UPLC BEH C18, 2.1 × 50 mm, 1.7 μm) was used. Protein precipitation method was used for detection: 50 μL plasma sample or brain sample was added with 200 μL acetonitrile containing an internal standard for precipitation and then vortexed for 3 min, and the supernatant was centrifuged in LC–MS/MS at 13000 rpm/min for 10 min.

### 2.11 Immunofluorescent Staining

Tumor tissue was fixed with 4% paraformaldehyde and immersed in 15% sucrose and 30% sucrose at 4°C overnight. Tumor tissue sections (thickness = 14 μm) were incubated with the blocking solution for 1 h at room temperature, followed by incubation with primary antibodies (MTAP [1:300] and PRMT5 [1:300]) at 4°C overnight. Then, secondary antibody (Alexa Fluor 488) and DAPI were incubated. The mounted slides were examined under a laser scanning confocal microscope (LSM 800; Zeiss, Wetzlar, Germany) to measure the fluorescence.

### 2.12 Statistical Analysis

All statistical analyses were performed using GraphPad Prism 8 Software (La Jolla, CA, USA). Data were expressed as mean ± SD. Statistical differences among groups were analyzed by using one‐way ANOVA, followed by Tukey's post hoc test.

## 3 Results

### 3.1 Clinicopathological Features Influencing Patients' Death and Recurrence

**FIGURE 1:**
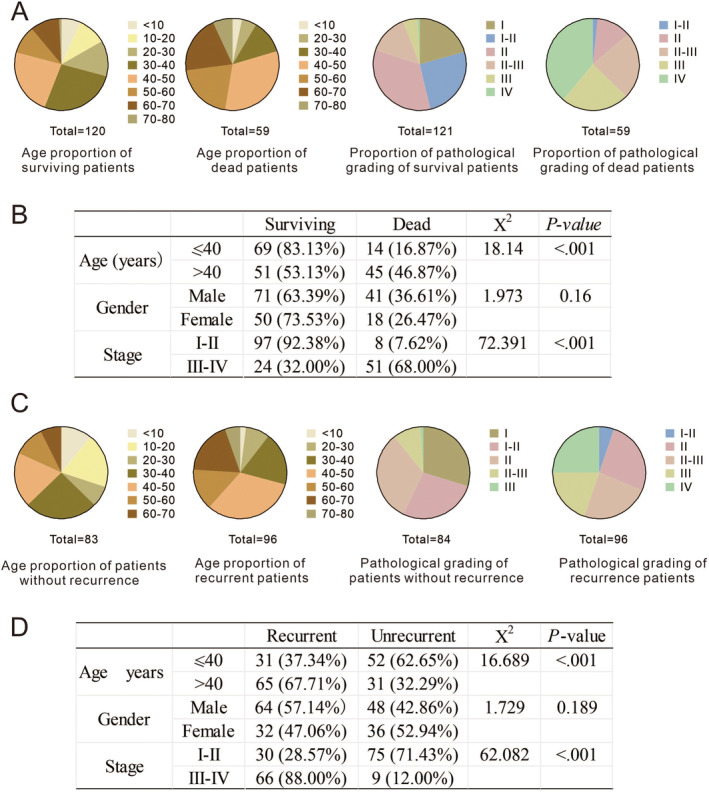
Clinicopathological features influencing patients' death and recurrence. The result of statistics based on the information in Supporting Information 1, and A and B is the factors affecting death, C and D is the factors affecting recurrence. Because a patient lacks age information, we only counted 179 people for age‐related content.

First, information of death and recurrence in 180 patients with GBM was statistically calculated. We found a relationship between patient age and GBM grade with survival and recurrence rates. As shown in Figure 1 and Supporting Information 1, higher age or pathological grade had a key role as a negative regulator affecting the patient's death and recurrence. As shown in Figure 1A,B, patients aged > 40 years had a mortality rate of 46.87%, which was significantly higher than those aged ≤ 40 years (16.87%, *p* < 0.001). Similarly, the higher the disease staging, the significantly higher the mortality rate of the patients (7.62% vs. 68.00%, *p* < 0.001). In terms of recurrence rate, the higher the age and grade of pathology, the greater the risk of recurrence (Figure 1C,D). No recurrence was found in patients with a pathologic classification of I (*n* = 25). Among 80 patients with a pathologic classification of II, 35 patients recurred (37.5%). The recurrence rate increases sharply in higher grades. In the III‐grade patients, the recurrence rate reached 82.35% (42 out of 51 patients recurred), while patients with a pathologic classification of IV had a 100% recurrence rate (*n* = 24). However, no statistically significant correlations were found between gender and patient's mortality or recurrence rate.

### 3.2 MTAP Deficiency Is Prevalent in GBMs and May Affect Tumor Grading

**FIGURE 2:**
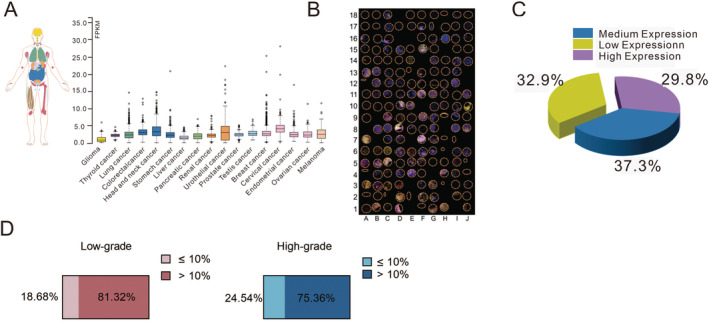
MTAP deficiency is ubiquity in cancer. (A) The data of low expression of MTAP in various cancer come from TCGA database. paraffin‐embedded tissue microarray of human glioma specimens. (B) Multiplexed immunofluorescence staining of paraffin‐embedded tissue microarray of human glioma specimens including MTAP, PRMT5, and Iba‐1 was shown. Blue, green, red, and yellow represent DAPI, MTAP, PRMT5, and Iba‐1 respectively. The statistics show that in C and D, C shows the expression of MTAP among patients, and D represents the proportion of low expression (≤ 10%) of MTAP in high and low pathological grades.

Previous studies have shown that MTAP loss is prevalent in GBM, and it occurs in half of all patients [20]. TCGA data in the database showed that deletion of MTAP is associated with multiple types of tumors (Figure 2A). To further analyze the expression of MTAP in tumor cells and its surrounding environment in GBM patients, paraffin‐embedded tissue microarray of human glioma specimens using multiplexed immunofluorescence staining was used to demonstrate MTAP, PRMT5, and microglia (Iba‐1) expression in glioma tissues (Figure 2B). Further, blue, green, red, and yellow represent DAPI, MTAP, PRMT5, and Iba‐1, respectively. Due to incomplete information on some patients, information on 161 of these 180 patients were statistical (Figure 2B and Supporting Information 2). As shown in Figure 2C, 53 patients (32.9%) had low expression (< 20%) of MTPA‐positive cells, while 60 (37.3%) had moderate (20% ~ 60%) and 48 (29.8%) had high (> 60%) expression. Staining results suggested that majority of BGMs have low levels of MTAP, which is consistent with previous conclusions.

Some studies have pointed out that the deletion of MTAP in different grades of glioma is different, and MTAP expression loss in the high‐grade glioma subgroup was almost twofold greater than in the lower‐grade glioma subgroup [21]. Further, in view of the potentially synthetic lethal effects resulting from MTAP deletion, MTAP expression was counted in low grades (grades I–II, 92 individuals) and high grades (grades III–IV, 69 individuals). We defined the expression below 10% as MTAP deletion, and our analysis showed that MTAP deletion in high‐grade glioma patients is more serious, although it is not twice as high (Figure 2D). The above results suggested that MTAP deficiency might increase in incidence with disease progression in GBM.

### 3.3 No Relationship Was Found Between the Expression of PRMT5 and MTAP Deletion

**FIGURE 3:**
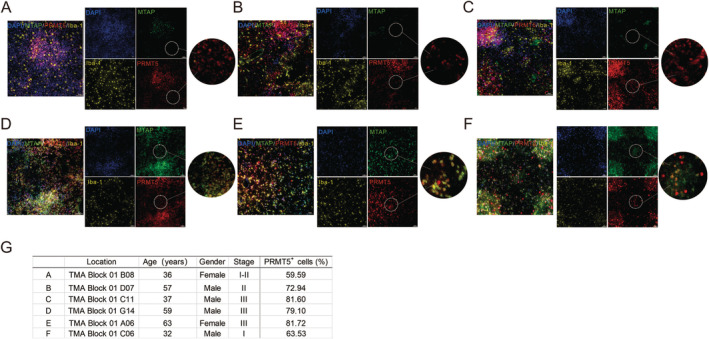
No relationship was found between the expression of PRMT5 and MTAP deletion. A–F are fluorescent staining information exhibits from different patients, and illustrates that the deletion of MTAP did not affect the expression of PRMT5. Where A–C represented the absence of MTAP, and D–F was the state when MTAP normally expressed. Its sample information (A–F) was presented by G.

PRMT5 is usually regarded as a marker of malignant progression in gliomas, and its expression increases simultaneously with malignant progression. PRMT5 is usually lowly expressed in control brain tissue and glial cells of low‐grade astrocytomas, while it is highly expressed in GBMs [16]. Biologically, MTAP is involved in the metabolism of MTA and regenerates methionine needed for S‐adenosyl‐L‐methionine (SAM) production. In the absence of MTAP, accumulated MTA acts as an intrinsic and selectively competitive inhibitor with a structure binding more readily to PRMT5, which reduces the binding of SAM to PRMT5 and in turn, limits PRMT5's methyltransferase activity [22]. Multiplexed immunofluorescence staining also reflected the expression of PRMT5 in glioma (Figure 2B). Therefore, it was next verified whether the deletion of MTAP affects the changes in PRMT5 expression by analyzing the staining. Staining of sections from three representative GBM patients with the deletion of MTAP expression (Figure 3A–C) and normal expression (Figure 3D–F) showed that deletion of MTAP did not affect the expression of PRMT5. The statistics of relevant patient information and percentage of PRMT5^+^ cells are shown in Figure 3G.

### 3.4 MTA Secreted by MTAP‐Deleted Cells May Be Metabolized by MTAP‐Expressing Cells Around the Tumors

**FIGURE 4:**
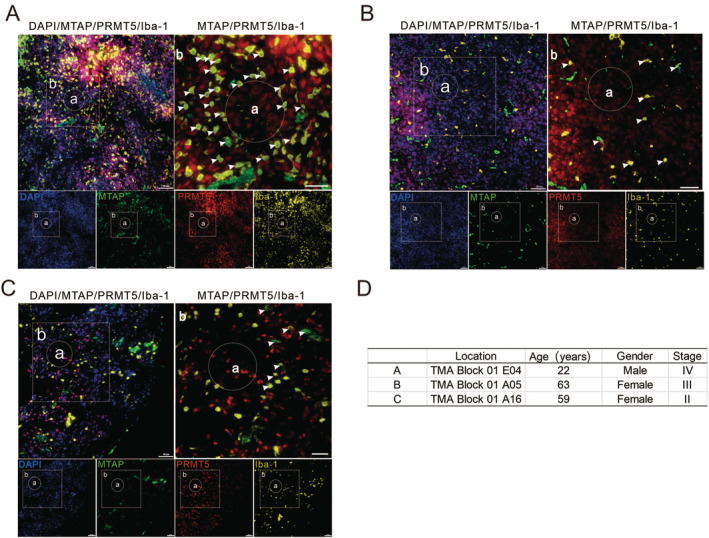
MTA secreted from MTAP‐deleted cells might be metabolized by MTAP‐expressing cells presented inside tumors. A–C showed that MTAP‐expressing microglia survived around MTAP‐deficient glioma cells, and the patient information of A–C samples is shown in D.

GBMs may have up to 75% (range 25% ~ 75%) nonmalignant stromal cells. Therefore, a homozygous MTAP‐deleted GBM tumor is an admixture of nonmalignant MTAP‐expressing stroma and MTAP‐null malignant glioma cells. The large amount of MTA synthesized by MTAP‐deficient tumor cells is constantly secreted outside the cell, resulting in much higher levels of extracellular MTA than intracellular [22]. However, if MTAP‐deficient tumor cells are surrounded by MTAP‐expressing normal cells, they may metabolize the secreted MTA, thus diminishing the synthetic lethal effect of MTAP‐deficient cells [9]. Therefore, when staining human GBM tissues, we not only performed MTAP and PRMT5 staining but also labeled microglia by Iba‐1 staining. As shown in Figure 4, Iba‐1‐positive cells surrounding MTAP‐deficient cells normally expressed MTAP, implying that MTA synthesized by MTAP‐deficient tumor cells might be metabolized by normal cells in the brain, and these findings were the same across patients of different ages, gender, and disease processes.

### 3.5 Efficacy of PRMT5/MTA Composition Inhibitor May Be Attenuated by the Presence of MTAP‐Expressing Normal Cells Around GBMs

**FIGURE 5:**
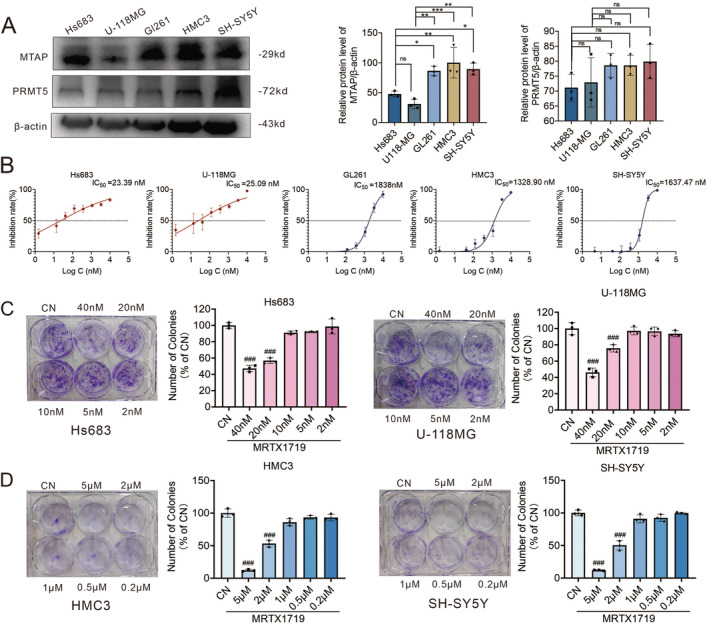
PRMT5/MTA inhibitor inhibited proliferation of MTAP‐deficient cells better than MTAP‐expressing normal cells. (A) Expression of MTAP and PRMT5 in glioma cells (Hs683, U‐118MG and GI261), microglia (HMC3), and neuronal (SH‐SY5Y) cells as determined by Western blot. *N* = 4, *p < 0.05, **p < 0.01, ***p < 0.001, ns indicates that there is no statistical difference. The inhibitory effect of MRTX1719 on the proliferation of the above five cell types were determined (B), and the colony diagrams and statistics of various cells under different concentrations of MRTX1719 are also shown (C and D). *N* = 3, ^###^ *p* < 0.001 versus CN group.

Having identified MTAP‐WT cells' presence inside MTAP‐deleted tumors, we sought to investigate whether the PRMT5/MTA composition inhibitor played an ideal role in the tumor environment. First, we determined the inhibitory effect of MRTX1719 on the growth and proliferation of MTAP‐deficient glioma cells (Hs683 and U‐118MG), MTAP‐normal glioma cells (GL261), MTAP‐normal microglial (HMC3), and neuronal cells (SH‐SY5Y) (Figure 5A). As shown in Figure 5B, the half‐maximal inhibitory concentration (IC_50_) of MRTX1719 in MTAP‐deleted glioma cells was significantly lower than that in MTAP‐normal cells, and such a situation will not be different because of different cell lines, which indicated that therapeutic targeting of PRMT5/MTA was a promising strategy to selectively inhibit the proliferation of MTAP‐deficient glioma cells. Colony formation results also demonstrated the same conclusion (Figure 5C,D).

**FIGURE 6:**
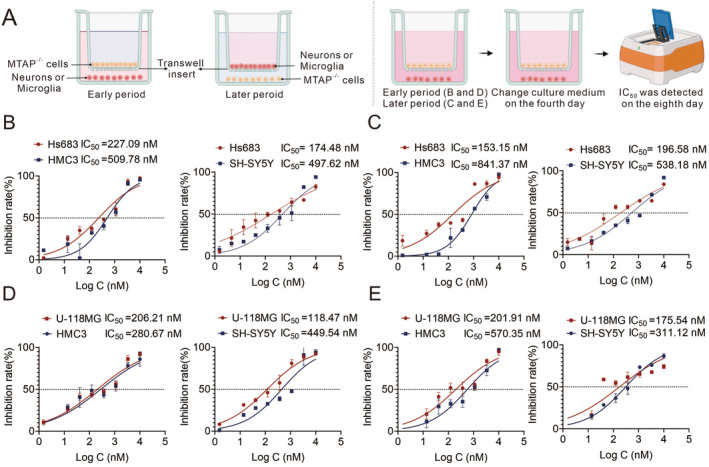
Inhibition of tumor proliferation of PRMT5/MTA composition inhibitor may be attenuated by the presence of stroma in the tumor microenvironment. To simulate the in vivo survival of cells with normal MTAP expression around MTAP‐deficient tumor cells, we used Transwell chambers to construct an in vitro model. The operation schematic diagram is shown in A. Due to the early stages of glioma existence, the tumors are smaller and there are more MTAP‐expressing normal cells in the brain surrounding them. More neurons or microglia inoculated in the lower layers and a smaller number of MTAP‐deficient glioma cells inoculated in Transwell chambers were used to represent the early stages of gliomas. Accordingly, a higher number of glioma cells were inoculated in the lower layer, while a smaller number of neurons or microglia were inoculated in the upper layer representing a dense stage of glioma progression to an advanced stage. The inhibitory effect of MRT1719 on the proliferation of glioma cells, microglia and neurons in the above inoculated states was determined separately (B–E). Compared with the IC_50_ of single cell detected in Figure 5, it was found that the IC_50_ of MRTX1719 on MTAP‐deficient cells increased and that on normal cells decreased.

Next, to simulate the growth of tumor cells mixed with normal brain cells in vitro, Transwell chambers were employed. As shown in Figure 6A, in order to simulate the early stage of tumor, we inoculated the normal expression cells of MTAP in the lower layer while the deletion cells of MTAP in Transwell chambers. When simulating the advanced stage of tumor, MTAP‐deficient cells were inoculated in the lower layer while MTAP‐normal expression cells in the upper layer. When MTAP‐deleted U‐118MG and Hs683 cells were co‐cultured with MTAP‐WT neuronal or microglia cells, the effect of MRTX1719 in inhibiting tumor cell proliferation was weakened significantly, and the lethality of normal brain cells was significantly enhanced (Figure 6B–D). The above results suggested that the inhibitory effect of MRTX1719 on the proliferation of MTAP‐deficient glioma cells might be attenuated due to the presence of MTAP‐expressing normal brain cells.

### 3.6 Antitumor Activity of PRMT5/MTA Composition Inhibitor Was Also Attenuated In Vivo

**FIGURE 7:**
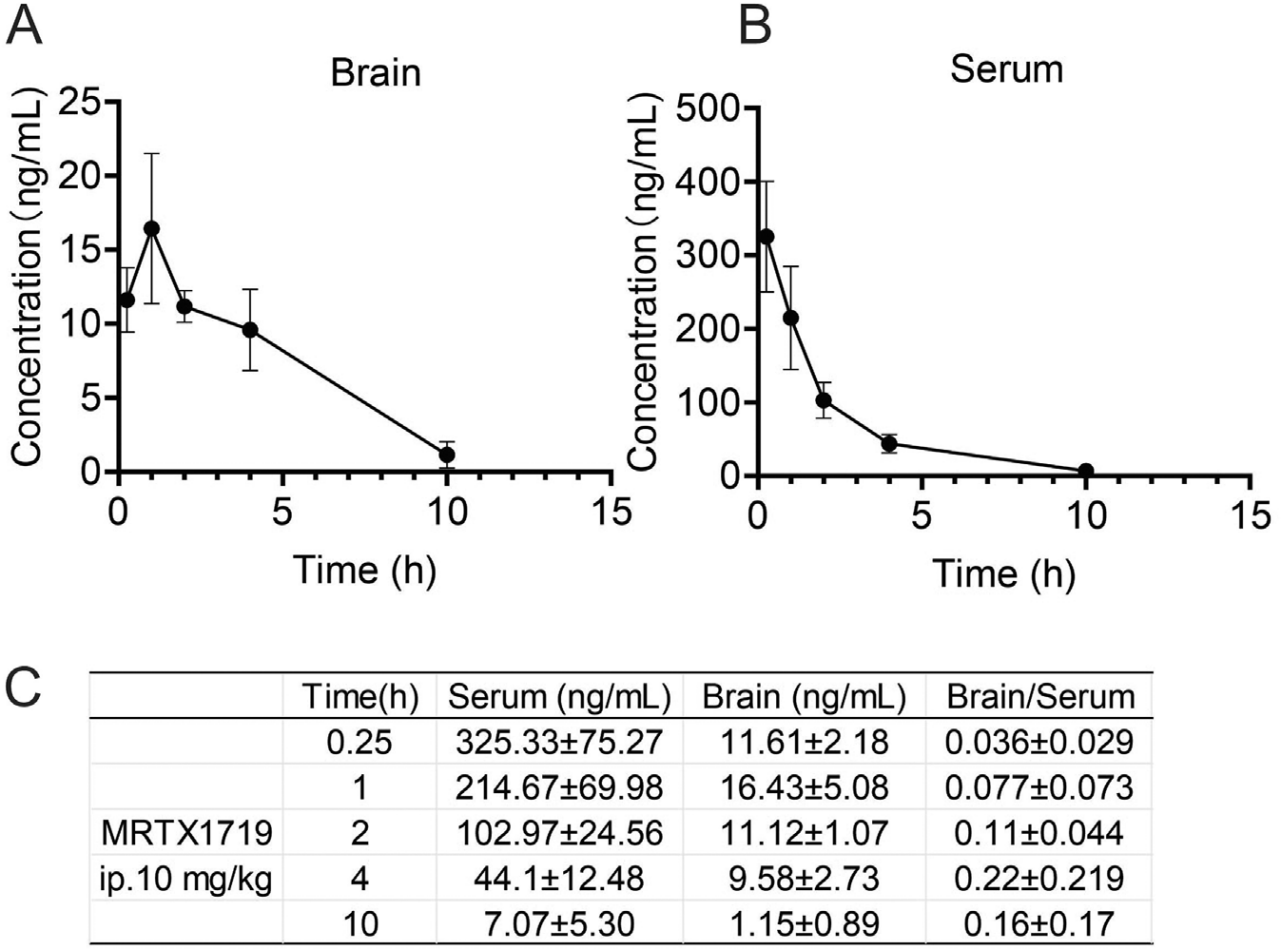
Detection of serum brain tissue distribution of MRTX1719. Concentration–time profiles of MRTX1719 in brain (A) and serum (B) (*n* = 3). Concentration ratio of MRTX1719 in the brain to plasma (C).

It is already well understood that cell culture conditions do not always accurately reflect physiological conditions. Therefore, we progressed on to verify the antitumor activity of MRTX1719 in vivo. First, we tested whether MRTX1719 crosses the blood–brain barrier. The results showed that the concentration of MRTX1719 in the brain reached the maximum value about 1 h after subcutaneous injection. The concentration ratio of MRTX1719 in the brain to plasma reached its maximum at 4 h of administration (Figure 7).

**FIGURE 8:**
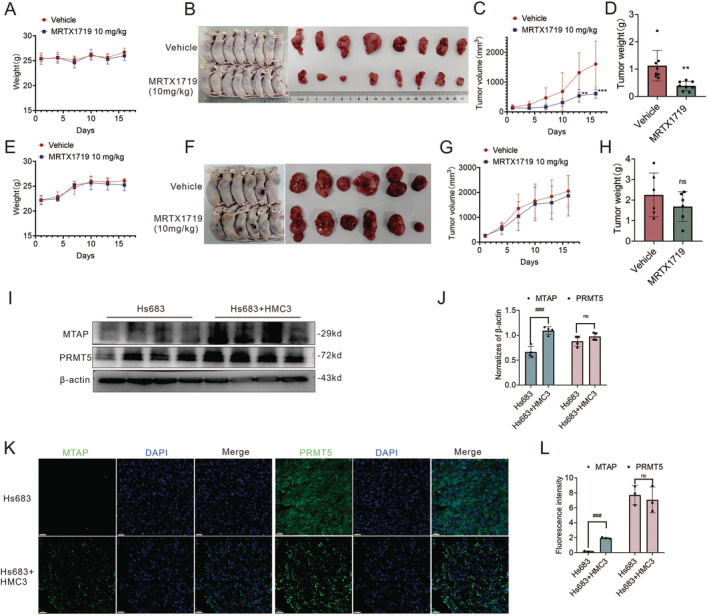
Antitumor activity of PRTX1719 inhibitory effect of MRTX1719‐mixed Hs683 and HMC3 graft tumors was weaker than Hs683 pure graft tumors. 10 mg/kg MRTX1719 significantly reduced the tumor size and weight of Hs683 pure graft tumors without affecting the mice weight (A–D). *N* = 8, **p < 0.01, ***p < 0.001 versus Vehicle group. However, 10 mg/kg MRTX1719 did not significantly reduce the volume and weight of gliomas (Hs683) and microglia (HMC3) mixed tumors (E–H). *N* = 6, ns indicates that there is no statistical difference. Expression of PRMT5 and MTAP in tumor tissues was detected using Western Blot (*n* = 4) and Fluorescence staining (*n* = 3, bar = 20 μm) in I–J and K–L individually, ^###^ *P* < 0.001.

**FIGURE 9:**
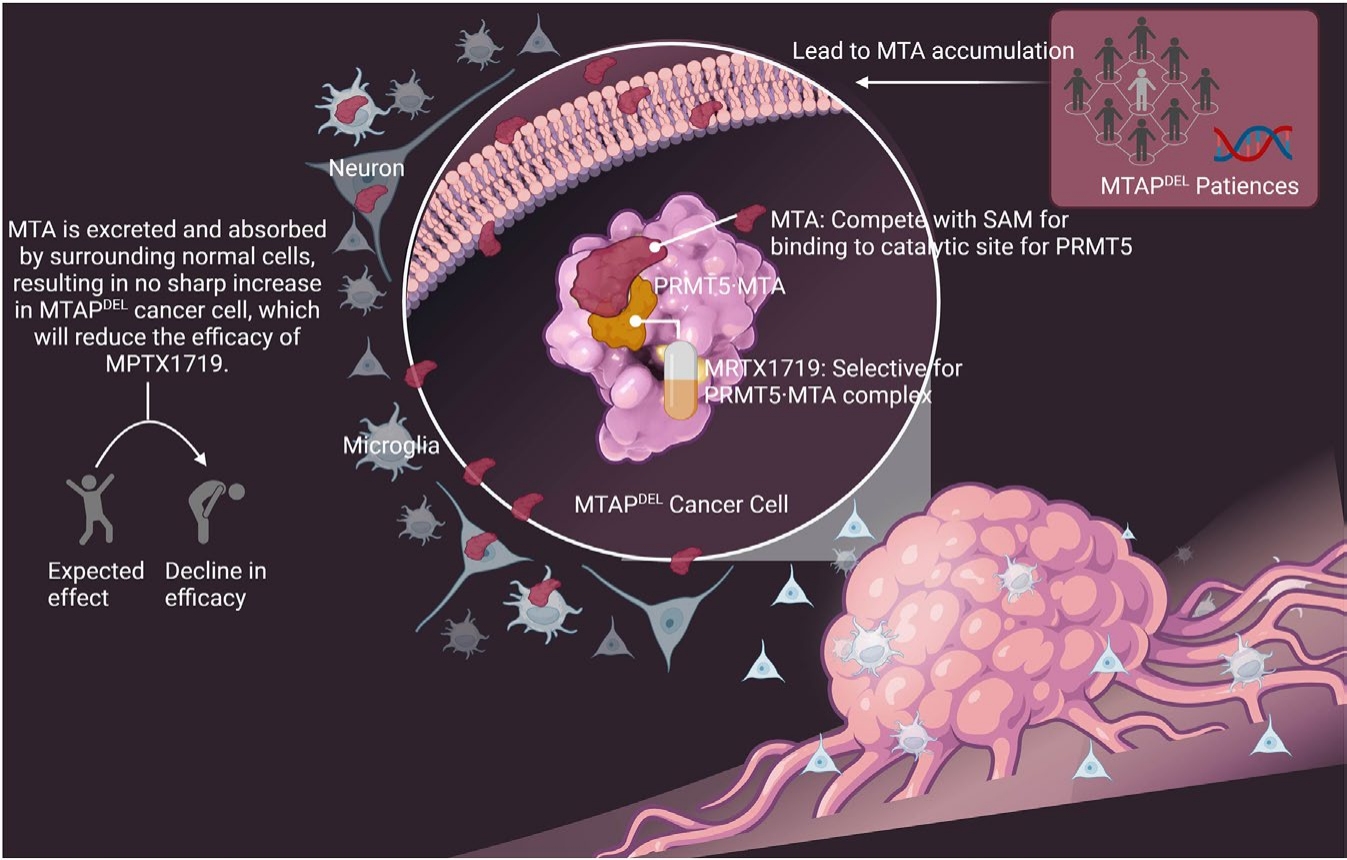
Schematic diagram.

Consistent with the data in vitro, MRTX1719 indeed reduced the Hs683‐transplanted tumor volume without reducing the body weight of the mice (Figure 8A–D). Notably, compared with the Hs683‐transplanted tumor, MRTX1719 had a lower ability to suppress the tumor volume in mixed transplanted tumors of Hs683 and HMC3 (Figure 8E–H). In addition, we evaluated the reliability of establishing the subcutaneous tumor model by Western blot and immunofluorescence (Figure 8I–L). In our data, it is confirmed that the subcutaneous tumor established by Hs683 has less expression of MTAP and hardly affects the expression of PRMT5 compared with the mixed subcutaneous tumor inoculated with Hs683 and HMC3. Our dataset proved that MTAP deletions might be leveraged as a point of selective vulnerability but gave pause to whether it would translate to the clinic. In addition, the application of MRTX1719 did not significantly affect the body weight of nude mice, representing the safety of its application. Therefore, our conclusion was that targeted therapies against PRMT5/MTA could be suitable candidates, but their ability to achieve suitable therapeutic effects in the clinic might require more in‐depth studies (Figure 9).

## 4 Discussion

GBM is the most aggressive glioma and the deadliest of all. Even with the current treatments, namely surgical resection, radiotherapy, and chemotherapy, it is still an incurable disease with a fairly poor survival rate. It is mainly present in adults, but can also occur in children, with a higher incidence in men compared to women [23, 24]. In addition to being highly invasive, GBM is still prone to recurrence because it grows rapidly in the brain and is resistant to chemotherapy [25]. In this study, we counted the basic information of 180 patients with gliomas, and the results showed that age and pathological grade are the main factors, but gender is not so important. The reason for this difference may be the insufficient sample size.

Recent advances in cancer metabolism research have identified molecular defects leading to vulnerabilities. Researchers found genomic deletions of tumor suppressor genes can involve adjacent metabolic genes, thus generating a targetable vulnerability [13]. Tumor suppressor genes lost via biallelic deletions cannot be directly targeted. however, cancers with tumor suppressor alterations may exhibit vulnerabilities that can be exploited for the treatment of cancer [14]. MTAP is a metabolic enzyme functioning in the purine/methionine salvage pathway. Normally, MTAP functions by metabolizing MTA, to eventually produce adenine and methionine, resulting in the salvage of these metabolites [16, 26]. Homozygous deletion of MTAP is frequently observed in cancers as it is an especially efficient mechanism for removing multiple tumor suppressor transcripts, including non‐small cell lung cancer (NSCLC), mesothelioma, pancreatic cancer, glioblastoma, head and neck cancer, esophageal cancer, bladder cancer, and malignant peripheral nerve sheath tumors (MPNST) [8, 27]. It is in our data that the proportion of MTAP expression less than 20% is very high, accounting for about 32.9%, which shows that MTAP deletion is common in glioma (Figures 1 and 2). No matter in the samples with low or high pathological grade, there is a certain proportion of MTAP deletion. More importantly, MTAP deletion is more likely to occur in high pathological grades.

Studies revealed that MTA, which accumulates in the context of MTAP loss, inhibited the activity of several enzymes, including PRMT5 [23]. PRMT5 is the major enzyme responsible for mono‐ and symmetric dimethylation of arginine. It transfers methyl groups from S‐adenosylmethionine to a guanidine nitrogen of protein arginine resulting in the reaction products methylarginine and S‐adenosylhomocysteine (SAH) [28]. Coincidentally, these data proved that SDMA modification in neoplastic cells from MTAP‐non‐expressing tumors was reduced by ∼50% compared with MTAP expressing tumors across tumor types. Those date all indicated that human cancers that do not express MTAP protein exhibit an apparent reduction of PRMT5 activity relative to MTAP‐expressing tumors [27]. In other words, MTAP deletion sensitizes tumor cells to PRMT5 inhibition. Meanwhile, MTAP deletion occurs in 50% of all GBM cases [16]. Therefore, synthetically lethal for the treatment of GBM cancers is promising. We found that the deletion of MTAP did not affect the expression of PRMT5 by multicolor immunofluorescence staining, which implied the feasibility of further targeting and inhibiting PRMT5 activity against MTAP‐deficient tumors (Figure 3).

Protein methylation on arginine residue was initially reported in late 1960s and early 1970s [29]. Among nine members of PRMT family, PRMT5, PRMT1, and CARM1 are most highly expressed in cancer. More seriously, high PRMT5 expression in human cancers is implicated in tumor promotion through histone tail modifications that repress target miRNAs and is correlated with worse prognosis of patients in a number of cancer types [30]. PRMT5 is important in normal tissue function, thus finding an inhibitor targeting PRMT5 directly is limited, but a synthetically lethal interaction with an MTAP‐negative background should be suitable [31]. Therefore, PRMT5 has recently emerged as a promising target in many cancers.

On the biology front, MTA, accumulated by MTAP loss, is biochemically determined to compete with SAM for binding to PRMT5 and is a moderately potent and selective inhibitor of PRMT5‐dependent deposition of SDMA on key protein substrates [8, 32]. There are SAM‐uncompetitive inhibitor of PRMT5 (EPZ015666/GSK3326595) that has been shown to be an effective antiproliferative agent in mantle cell lymphoma models with of PRMT5 overexpression [33]. However, EPZ015666 did not show substantial antiproliferative effect in MTAP‐deficient cell lines due to increased levels of MTA outcompeting SAM binding [32]. The higher affinity for MTA than SAM of PRMT5 means that MTA as a potent may provide a more excellent starting point for synthetic lethal [34, 35]. At the same time, high levels of MTA act as an endogenous inhibitor of PRMT5 activity, and sensitized cells to reduced levels of PRMT5. With chemical mastery, we can develop a small molecule that selectively binds to PRMT5 only in the presence of MTA killing tumor cells while sparing healthy ones, which may represent a synthetic lethal‐based precision medicine for patients with these cancers [36].

Therefore, MRTX1719, a new class of MTA‐cooperative PRMT5 inhibitors, came into being and overcame prior issues of drug toxicity. MRTX1719 demonstrated antitumor activity and inhibition of PRMT5‐dependent SDMA modification in MTAP‐deleted tumors xenografts, mouse or human hematopoietic cells [8]. Most importantly, it demonstrated that PRMT5 is a bona fide target in solid tumors [18]. Therapeutic targeting of PRMT5 in homozygous MTAP‐deleted cancer cells has thus been considered a promising strategy to selectively killed cancer cells, which predicated on the presence of exceedingly high MTA levels in MTAP‐deleted cancer cells compared to MTAP‐intact tissues. However, it is regretful that highly elevated MTA levels found in MTAP‐deleted glioma cell lines in culture cannot be extrapolated to primary GBMs. Large amounts of MTA produced by MTAP‐deficient gliomas may be taken up and metabolized by surrounding tissues with normal MTAP expression. In this case, the efficacy of PRMT5/MTA inhibitor MRTX1719 may not reach the expectation. To verify this speculation, we performed multicolor immunofluorescence staining of brain glioma sections with different disease grades. The results showed that MTAP‐deficient glioma tissues were surrounded by a large number of microglia with normal MTAP expression (Figures 3 and 4). This result is similar to previous studies [9].

Meanwhile, we used in vivo and in vitro glioma models to analyze whether this finding affects the antitumor activity of MRTX1719. MRTX1719 inhibits proliferation significantly in MTAP‐deficient gliomas compared to MTAP‐expressing normal gliomas. Meanwhile, MRTX1719 inhibited microglia and neuronal proliferation with IC_50_ up to μM level (Figure 5). To mimic the in vivo environment, we used MTAP‐deficient glioma cells co‐cultured with microglia and neurons. The results showed that the inhibitory effect of MRTX1719 on MTAP‐deficient gliomas was significantly attenuated, regardless of whether the proportion of microglia or neurons was at a high or low level (Figure 6). These showed that the decline of drug efficacy is probably due to the decomposition of MTA by surrounding cells that normally express MTAP, which led to the fact that the MTA content does not increase significantly in cells.

In order to further verify this conclusion, we attempted to determine the antitumor activity of MRTX1719 using an orthotopic in vivo model, but it is failed due to the specificity of MTAP‐deficient gliomas. However, to analyze the feasible role of MRTX1719 in the treatment of gliomas, its ability to penetrate the blood–brain barrier and its ability to inhibit subcutaneous tumor proliferation were determined. Our results showed that subcutaneous injection of MRTX1719 for about 1 h reached the maximum concentration in the brain. The concentration ratio of MRTX1719 in the brain to plasma reached its maximum at 4 h of administration. To mimic the environment of gliomas in vivo, mixed tumors of microglia and MTAP‐deficient gliomas were inoculated subcutaneously. Results of Western blot and immunofluorescence suggested that normal MTAP‐expressing tissues in the mixed‐inoculated tumor tissues were present in close proximity to the MTAP‐deficient tumor cells, as compared to MTAP‐deficient glioma cells inoculated alone. This result suggested that our subcutaneous tumor model is somewhat representative of the in vivo situation. Compared with nude mice implanted with Hs683 cells (MTAP‐deficient), the efficacy of MRTX1719 in mixed cell group will decrease, which is consistent with the conclusion of experiments in vitro.

## 5 Conclusions

As Yasaman said, the expedient translation of the MTAP‐deletion‐targeted precision therapies to the clinic is controversial [8]. This conclusion is also effectively illustrated by our data in vivo and in vitro. We also proved that the deletion of MTAP was common in GBM patients and also found that MTAP deletion did not affect the expression of PRMT5, as well as the expression of MTAP in surrounding cells. We do not completely rule out that MAT2A and/or PRMT5 inhibitors could prove useful in oncology. Only whether it can be widely used in clinic needs further attention.

## Author Contributions

**Yunjie Wang:** conceptualization (equal), methodology (equal), writing – original draft (equal), writing – review and editing (equal). **Xiaohui Sun:** conceptualization (equal), data curation (equal), investigation (equal), methodology (equal), project administration (equal), writing – original draft (equal), writing – review and editing (equal). **Runchen Ma:** data curation (equal). **Xiaofan Zhang:** data curation (equal). **Shengmin Ji:** data curation (equal). **Zhaofeng Liu:** data curation (equal). **Gangqiang Yang:** conceptualization (equal). **Hongbo Wang:** supervision (equal). **Peng Zhang:** funding acquisition (equal), project administration (equal). **Jianzhao Zhang:** investigation (equal), methodology (equal). **Jingwei Tian:** investigation (equal), project administration (equal), resources (equal).

## Ethics Statement

The animal study was reviewed and approved by the Ethics Committee of Yantai University.

## Conflicts of Interest

The authors declare no conflicts of interest.

## Supporting information


Supporting Information 1.

Supporting Information 2.

Supporting Information 3.

## Data Availability

All datasets generated for this study are included in the article/Supporting Information.
