# Supplementary material for: Inhibitory Effect of PRMT5/MTA Inhibitor on MTAP‐Deficient Glioma May Be Influenced by Surrounding Normal Cells
Source: Cancer Med. 2024 Dec 23;13(24):e70526. doi: 10.1002/cam4.70526 (PMC11664235; doi:10.1002/cam4.70526)
Supplement: Supplementary file 1 — Supporting Information 1. [file CAM4-13-e70526-s002.docx]

Table1 Patient information

| Sample | Gender | Age（years） | Stage | Description | |
| --- | --- | --- | --- | --- | --- |
| P01A0506 | Female | 37 | II | Survive | Unrecurrent |
| P01A0507 | Female | 20 | I | Survive | Unrecurrent |
| P01A0508 | Male | 44 | I-II | Survive | Unrecurrent |
| P01A0662 | Female | 2 | IV | Dead | Recurrent |
| P01A0510 | Female | 63 | III | Survive | Recurrent |
| P01A0509 | Male | 42 | I-II | Survive | Unrecurrent |
| P01A0663 | Male | 53 | IV | Dead | Recurrent |
| P01A0511 | Male | 69 | I-II | Survive | Unrecurrent |
| P01A0512 | Female | 11 | I | Survive | Unrecurrent |
| P01A0513 | Male | 16 | I-II | Survive | Unrecurrent |
| P01A0514 | Male | 41 | I-II | Survive | Unrecurrent |
| P01A0664 | Male | 24 | IV | Dead | Recurrent |
| P01A0515 | Male | 17 | II-III | Survive | Unrecurrent |
| P01A0516 | Female | 47 | II | Survive | Unrecurrent |
| P01A0517 | Female | 34 | I | Survive | Unrecurrent |
| P01A0518 | Female | 59 | II | Survive | Unrecurrent |
| P01A0519 | Female | 56 | II-III | Dead | Recurrent |
| P01A0520 | Female | 6 | I | Survive | Unrecurrent |
| P01A0521 | Male | 80 | II | Survive | Recurrent |
| P01A0522 | Female | 28 | I-II | Survive | Unrecurrent |
| P01A0523 | Male | 62 | II-III | Survive | Unrecurrent |
| P01A0524 | Male | 66 | II | Dead | Recurrent |
| P01A0525 | Female | 55 | I-II | Survive | Unrecurrent |
| P01A0526 | Female | 46 | I-II | Survive | Unrecurrent |
| P01A0527 | Female | 30 | I | Survive | Recurrent |
| P01A0528 | Male | 29 | III | Survive | Recurrent |
| P01A0529 | Female | 36 | I-II | Survive | Unrecurrent |
| P01A0530 | Female | 49 | II | Survive | Recurrent |
| P01A0531 | Male | 61 | II | Survive | Unrecurrent |
| P01A0532 | Male | 44 | II | Survive | Recurrent |
| P01A0533 | Male | 36 | II | Survive | Unrecurrent |
| P01A0534 | Male | 39 | II-III | Survive | Recurrent |
| P01A0535 | Male | 56 | II | Survive | Unrecurrent |
| P01A0536 | Male | 12 | I | Survive | Unrecurrent |
| P01A0537 | Male | 66 | I-II | Survive | Unrecurrent |
| P01A0538 | Female | 37 | I | Survive | Unrecurrent |
| P01A0539 | Male | 4 | I | Survive | Unrecurrent |
| P01A0540 | Male | 8 | I | Survive | Unrecurrent |
| P01A0541 | Male | 6 | I-II | Survive | Unrecurrent |
| P01A0542 | Male | 5 | I | Survive | Unrecurrent |
| P01A0543 | Female | 43 | II-III | Survive | Recurrent |
| P01A0545 | Male | 32 | I | Survive | Unrecurrent |
| P01A0544 | Male | 57 | II | Survive | Unrecurrent |
| P01A0546 | Female | 55 | III | Dead | Recurrent |
| P01A0547 | Female | 61 | I | Survive | Unrecurrent |
| P01A0548 | Male | 34 | II | Survive | Unrecurrent |
| P01A0549 | Male | 37 | III | Survive | Recurrent |
| P01A0550 | Male | 44 | II | Survive | Recurrent |
| P01A0665 | Male | 37 | IV | Dead | Recurrent |
| P01A0552 | Male | 46 | II | Survive | Unrecurrent |
| P01A0551 | Female | 52 | II | Survive | Unrecurrent |
| P01A0553 | Male | 20 | I-II | Survive | Unrecurrent |
| P01A0554 | Male | 60 | II | Survive | Recurrent |
| P01A0555 | Female | 41 | III | Dead | Recurrent |
| P01A0557 | Male | 41 | II | Survive | Unrecurrent |
| P01A0558 | Male | 68 | II-III | Survive | Recurrent |
| P01A0559 | Male | 31 | II | Survive | Recurrent |
| P01A0560 | Male | 63 | II | Dead | Recurrent |
| P01A0561 | Male | 65 | I-II | Survive | Recurrent |
| P01A0562 | Female | 57 | II-III | Dead | Recurrent |
| P01A0563 | Male | 57 | II | Survive | Recurrent |
| P01A0565 | Male | 47 | I-II | Survive | Recurrent |
| P01A0566 | Female | 42 | I-II | Survive | Unrecurrent |
| P01A0568 | Male | 60 | I-II | Survive | Recurrent |
| P01A0567 | Male | 31 | I | Survive | Unrecurrent |
| P01A0569 | Male | 63 | II | Survive | Recurrent |
| P01A0570 | Male | 20 | I-II | Survive | Unrecurrent |
| P01A0571 | Male | 69 | II | Dead | Recurrent |
| P01A0572 | Female | 42 | II | Survive | Recurrent |
| P01A0573 | Female | 26 | I | Survive | Unrecurrent |
| P01A0574 | Female | 41 | III | Dead | Recurrent |
| P02A0003 | Male | 49 | II-III | Survive | Unrecurrent |
| P01A0666 | Male | 41 | IV | Dead | Recurrent |
| P01A0667 | Male | 44 | IV | Dead | Recurrent |
| P01A0575 | Female | 36 | II | Survive | Unrecurrent |
| P01A0668 | Male | 22 | IV | Dead | Recurrent |
| P01A0576 | Male | 20 | II-III | Survive | Recurrent |
| P01A0577 | Male | 36 | I-II | Survive | Unrecurrent |
| P01A0578 | Female | 23 | II | Survive | Recurrent |
| P01A0579 | Female | 18 | I-II | Survive | Unrecurrent |
| P01A0580 | Female | 42 | II-III | Survive | Unrecurrent |
| P01A0581 | Male | 41 | II | Dead | Recurrent |
| P01A0582 | Male | 58 | II-III | Dead | Recurrent |
| P01A0583 | Male | 37 | II-III | Dead | Recurrent |
| P01A0584 | Female | 23 | I | Survive | Unrecurrent |
| P01A0585 | Male | 35 | II-III | Survive | Unrecurrent |
| P01A0669 | Female | 43 | IV | Dead | Recurrent |
| P01A0586 | Male | 40 | I-II | Survive | Recurrent |
| P01A0587 | Male | 46 | II-III | Dead | Recurrent |
| P01A0588 | Female | 17 | I-II | Survive | Unrecurrent |
| P01A0589 | Female | 47 | II-III | Survive | Recurrent |
| P01A0590 | Male | 33 | II-III | Survive | Unrecurrent |
| P01A0591 | Female | 37 | II-III | Dead | Recurrent |
| P01A0592 | Male | 42 | II-III | Dead | Recurrent |
| P01A0593 | Male | 4 | II | Survive | Unrecurrent |
| P01A0594 | Male | 79 | I-II | Dead | Recurrent |
| P01A0595 | Male | 74 | II | Dead | Recurrent |
| P01A0596 | Male | 50 | I-II | Survive | Unrecurrent |
| P01A0597 | Female | 36 | I-II | Survive | Unrecurrent |
| P01A0598 | Male | 32 | II | Survive | Recurrent |
| P01A0599 | Male | 36 | III | Survive | Recurrent |
| P01A0600 | Male | 38 | I | Survive | Unrecurrent |
| P01A0601 | Female | 18 | I | Survive | Unrecurrent |
| P01A0602 | Male | 3 | I-II | Survive | Unrecurrent |
| P01A0603 | Female | 42 | II | Survive | Recurrent |
| P01A0604 | Female | 45 | II-III | Dead | Recurrent |
| P01A0605 | Female | 52 | II | Survive | Recurrent |
| P01A0606 | Male | 20 | II-III | Survive | Unrecurrent |
| P01A0607 | Male | 68 | II | Dead | Recurrent |
| P01A0670 | Male | 60 | IV | Dead | Recurrent |
| P01A0608 | Female | 33 | III | Dead | Recurrent |
| P01A0671 | Female | 65 | IV | Dead | Recurrent |
| P01A0609 | Female | 14 | I | Survive | Unrecurrent |
| P01A0610 | Male | 28 | I-II | Survive | Unrecurrent |
| P01A0611 | Male | 37 | I-II | Survive | Unrecurrent |
| P01A0672 | Female | 60 | IV | Dead | Recurrent |
| P01A0612 | Female |  | I-II | Survive | Unrecurrent |
| P01A0613 | Male | 36 | II | Survive | Unrecurrent |
| P01A0614 | Female | 49 | I | Survive | Unrecurrent |
| P01A0615 | Male | 41 | III | Dead | Recurrent |
| P01A0616 | Male | 19 | II | Survive | Unrecurrent |
| P01A0617 | Male | 59 | III | Dead | Recurrent |
| P01A0618 | Male | 41 | I-II | Survive | Unrecurrent |
| P01A0673 | Male | 56 | IV | Dead | Recurrent |
| P01A0619 | Male | 58 | II-III | Dead | Recurrent |
| P01A0620 | Female | 40 | III | Dead | Recurrent |
| P01A0621 | Male | 33 | III | Survive | Unrecurrent |
| P01A0622 | Male | 35 | II-III | Dead | Recurrent |
| P01A0623 | Female | 38 | I | Survive | Unrecurrent |
| P01A0624 | Male | 47 | III | Survive | Recurrent |
| P01A0625 | Male | 34 | II-III | Survive | Recurrent |
| P01A0627 | Female | 52 | I | Survive | Unrecurrent |
| P01A0674 | Male | 46 | IV | Dead | Recurrent |
| P01A0628 | Female | 58 | II-III | Dead | Recurrent |
| P02A0004 | Female | 59 | II-III | Survive | Unrecurrent |
| P01A0629 | Female | 30 | III | Dead | Recurrent |
| P01A0675 | Male | 37 | IV | Survive | Recurrent |
| P01A0676 | Male | 65 | IV | Dead | Recurrent |
| P01A0677 | Male | 9 | IV | Dead | Recurrent |
| P01A0678 | Male | 41 | IV | Dead | Recurrent |
| P01A0679 | Male | 49 | IV | Dead | Recurrent |
| P01A0630 | Male | 49 | III | Dead | Recurrent |
| P01A0680 | Male | 78 | IV | Dead | Recurrent |
| P01A0631 | Female | 48 | II | Survive | Recurrent |
| P01A0633 | Male | 36 | II | Survive | Recurrent |
| P01A0634 | Male | 40 | I-II | Survive | Unrecurrent |
| P01A0635 | Male | 41 | II | Survive | Recurrent |
| P01A0636 | Female | 45 | II | Survive | Unrecurrent |
| P01A0687 | Female | 6 | I | Survive | Unrecurrent |
| P01A0637 | Male | 54 | I-II | Survive | Unrecurrent |
| P01A0638 | Female | 32 | II | Survive | Unrecurrent |
| P01A0639 | Male | 48 | II | Survive | Unrecurrent |
| P01A0640 | Male | 30 | III | Dead | Recurrent |
| P01A0641 | Female | 24 | I-II | Survive | Unrecurrent |
| P01A0642 | Male | 52 | II-III | Dead | Recurrent |
| P01A0643 | Female | 26 | II-III | Survive | Recurrent |
| P01A0681 | Male | 62 | IV | Dead | Recurrent |
| P01A0644 | Male | 56 | II | Survive | Unrecurrent |
| P01A0645 | Male | 15 | II | Survive | Unrecurrent |
| P01A0682 | Male | 67 | III | Dead | Recurrent |
| P01A0646 | Male | 30 | I | Survive | Unrecurrent |
| P01A0647 | Female | 70 | II-III | Dead | Recurrent |
| P01A0648 | Female | 36 | II-III | Survive | Recurrent |
| P01A0649 | Male | 41 | II | Dead | Recurrent |
| P01A0683 | Male | 63 | IV | Dead | Recurrent |
| P01A0684 | Male | 45 | IV | Dead | Recurrent |
| P01A0650 | Male | 37 | II | Survive | Unrecurrent |
| P01A0651 | Female | 69 | III | Dead | Recurrent |
| P01A0653 | Female | 19 | II | Survive | Unrecurrent |
| P01A0652 | Male | 49 | II | Survive | Unrecurrent |
| P01A0654 | Female | 66 | I | Survive | Unrecurrent |
| P01A0685 | Male | 41 | IV | Dead | Recurrent |
| P01A0655 | Male | 56 | III | Dead | Recurrent |
| P01A0656 | Female | 20 | I | Survive | Unrecurrent |
| P01A0657 | Female | 28 | II | Survive | Recurrent |
| P01A0658 | Male | 57 | III | Dead | Recurrent |
| P01A0659 | Male | 10 | I | Survive | Unrecurrent |
| P01A0686 | Female | 28 | IV | Dead | Recurrent |
| P01A0660 | Female | 36 | II-III | Survive | Recurrent |
| P01A0661 | Male | 40 | II-III | Dead | Recurrent |
