# Supplementary material for: Inhibitory Effect of PRMT5/MTA Inhibitor on MTAP‐Deficient Glioma May Be Influenced by Surrounding Normal Cells
Source: Cancer Med. 2024 Dec 23;13(24):e70526. doi: 10.1002/cam4.70526 (PMC11664235; doi:10.1002/cam4.70526)
Supplement: Supplementary file 3 — Supporting Information 3. [file CAM4-13-e70526-s001.docx]

Serum：10h

4h

2h

1h

15min

Brain：10h

4h

2h

1h

15min
